# Human–Environment Interactions in GeoHealth: Addressing Terrestrial Ecosystem Health, Land Degradation, and Carbon Management

**DOI:** 10.1029/2025GH001718

**Published:** 2026-01-28

**Authors:** Baijun Shang, Ranjay K. Singh, Yingui Cao, Tong Li

**Affiliations:** ^1^ Center for Agricultural Resources Research Institute of Genetics and Developmental Biology Chinese Academy of Sciences Shijiazhuang China; ^2^ Division of Agricultural Extension Indian Council of Agricultural Research New Delhi India; ^3^ School of Land Science and Technology China University of Geosciences Beijing China; ^4^ Key Lab of Land Consolidation and Rehabilitation Ministry of Natural Resources Beijing China; ^5^ Observation and Research Station of Land Reclamation in Loess Plateau Mining Area Ministry of Natural Resources Beijing China; ^6^ School of Agriculture and Food Sustainability The University of Queensland St Lucia QLD Australia

**Keywords:** GeoHealth, terrestrial ecosystem, human–environment interaction, land degradation, soil carbon, sustainable management

## Abstract

Global environmental changes have posed threats to ecosystems worldwide. Safeguarding terrestrial ecosystem health in particular is fundamental to achieving global sustainability targets, yet land degradation, carbon depletion and climate extremes continue to undermine resilience due to climate change and human activities. Therefore, Understanding human‐environment interactions is increasingly important for enhancing the resilience of terrestrial ecosystems under global change. The collection for this special issue addresses urgent challenges of land degradation, soil carbon loss, and ecosystem vulnerability by assembling eight regionally grounded studies from diverse landscapes of Asia. Collectively, these contributions reveal how land‐use transitions, restoration strategies and climate variability shape ecosystem health and carbon dynamics, while advancing methodological and governance frameworks that link science with policy. The collection offers critical insights and practical lessons for scholars and policy planners to sustainably manage land resources within the GeoHealth paradigm.

## Plain Language Summary

Human activities and climate change are putting stress on forests, grasslands and farmlands. These pressures lead to land degradation, soil carbon loss and reduced ability of ecosystems to provide food, water, and clean air. This special issue brings together eight studies from Asia that examine how land use, restoration, and climate change affect soil carbon and ecosystem health. The findings show that solutions must be tailored to local conditions, for example, grazing strategies differ across grasslands, and restoration success depends on soil type. Urban expansion creates trade‐offs between food and water security, while forests in arid regions are especially at risk. The studies highlight that sustainable management needs both science‐based approaches and strong governance.

## Key Points

- Changes in land use patterns and restoration practices are central drivers of terrestrial ecosystem health and soil carbon dynamics
- Ecosystem responses are context‐dependent, requiring site‐specific and scale‐appropriate approaches and interventions
- Integrating biophysical, socio‐economic and institutional factors creates cross‐sector pathways for sustainable management

## 1 Introduction

Human‐environment interactions have become one of the critical research frontiers in the Anthropocene (Soga & Gaston, 2021), as terrestrial ecosystems face escalating pressures from population growth, rapid urbanization and intensified land use (Gibbard et al., 2022; Zhao et al., 2025). According to a statistics, the global population will reach 9.8 billion by 2050, and food demand driven by population expansion has led to a continuous increase in cultivated land area, while the proportion of urbanized residents has soared from 10% (in 1900) to 75% (in 2050). The expansion of cultivated land area and urbanization development may lead to the fragmentation of ecosystems such as forests, grasslands and wetlands; and disrupt the original ecological balance (Zhang et al., 2025).

These ecosystems provide indispensable services‐including carbon sequestration, water regulation, food production, and biodiversity conservation‐that underpin both environmental stability and human well‐being (Cui et al., 2025; He et al., 2025; Mandle et al., 2020; Yang et al., 2023). From the perspective of regulating services, forests are important “carbon sink” for mitigating climate change (Liu et al., 2024) and the existing forests in 2020 contribute the most to the future total carbon sink (Leng et al., 2024). Furthermore, forests can reduce the risk of flood disasters by regulating precipitation patterns(Singh et al., 2025). From the perspective of provisioning services, ecosystems directly provide material products and resources for humans.

Yet, widespread land degradation, unsustainable agricultural practices and climate extremes are accelerating the decline of ecosystem health and resilience, undermining progress toward global sustainability goals, such as the United Nations Sustainable Development Goals (SDGs) and international carbon neutrality commitments (Bardgett et al., 2021; Li et al., 2023, 2025). Despite substantial advances in ecological and environmental sciences (Fraisl et al., 2022), significant knowledge gaps remain regarding how human activities interact with ecosystem processes, how land use dynamics influence soil carbon and ecosystem resilience; and how science‐based evidence aligned with grassroots insights can better inform policies for sustainable land management (He et al., 2026). Addressing these gaps is essential not only for safeguarding terrestrial ecosystem health, but also for strengthening the broader human‐environment nexus under the emerging GeoHealth framework.

## 2 Scope of the Special Collection

This special collection was conceived to respond to the urgent global environmental challenges by fostering interdisciplinary dialog across ecology, geography, soil science, human dimensions and environmental policy. The collection brings together eight research articles that not only provide regionally grounded evidence‐from the Yangtze River Delta and Beijing–Tianjin–Hebei to the Qinghai–Tibetan Plateau, Central Asia and beyond, but also offer innovative methodological frameworks and approaches for linking human activities with ecosystem processes. Collectively, these contributions address pressing gaps identified on ground and in global literature: they reveal how urbanization shapes trade‐offs between food and water security, how crop rotation and grazing management alter soil carbon dynamics, how forest resilience is challenged under increasing aridity and how planetary boundaries can be operationalized for sustainability assessment in arid regions (Rockström et al., 2024). By situating these studies within the broader GeoHealth framework, the Special Issue advances an integrated understanding of how human–environment interactions can be assessed, managed, and translated into actionable science–policy pathways.

## 3 Thematic Contributions in This Collection

This section synthesizes case studies that explore the impact of human activities, including land‐use change, grazing and urbanization on ecosystem processes such as soil carbon dynamics, grassland restoration, forest resilience and urban environmental equity.

*Land use transitions and ecosystem services:* Three studies emphasize the tensions between human development and ecosystem functions in agroecosystems. The study of the Yangtze River Delta identified how rapid urbanization intensified trade‐offs between food security and water quality, though it also revealed opportunities for “win–win” outcomes (Xue et al., 2025). This indicated the potential implications of land use change in a given landscape or ecosystem. The application of a novel coordination framework in the Beijing–Tianjin–Hebei–Inner Mongolia region revealed a widespread imbalance between human activity intensity and ecosystem service provision (Fang et al., 2025). This has drawn attention to plan and execute sustainable activities with a principle to maintain human‐ecosystem equilibrium. In southeastern China, researchers demonstrated that integrating historical crop rotation data into soil organic matter (SOM) mapping improved the prediction accuracy of SOM (Zhou et al., 2025), a result that underscores the critical role of land‐use legacies in sustaining soil fertility and enhancing soil carbon storage.

*Grassland management and restoration:* Two articles focus on the Qinghai–Tibetan Plateau, highlighting grasslands as both vulnerable and critical for carbon cycling. One study showed that seasonal versus continuous grazing regimes differentially affect soil carbon mineralization, with soil properties outweighing microbial factors in regulating carbon loss (Cao et al., 2025). Therefore, this leads to an insight that there is a need to optimize the inter‐seasonal and inter‐annual grazing patterns in order to enhance the sustainability of the grazing ecosystem. The other study revealed that active restoration enhances sub‐soil carbon storage in nutrient‐poor soils, whereas passive restoration better conserves carbon in carbon‐rich topsoil (Wu et al., 2025). Together, these findings stress the need to align restoration strategies with site‐specific soil carbon baselines. This also necessitates to consider the role of restoration practices in location specific management of stressful soils (water and wind eroded soils, saline and sodic soils and acid soils) in view of Land Degradation Neutrality (LDN) while achieving SDG‐15.

*Forest resilience and regional sustainability*: Another cluster examines forest ecosystems and regional thresholds of sustainability. Across China, increasing aridity and precipitation variability have driven resilience declines in nearly half of restored forests, especially where species richness is low, or forestation history is short (Wang et al., 2025). At a broader scale, a downscaled planetary boundaries framework applied in Central Asia showed that land‐use intensity in Kazakhstan and Uzbekistan exceeds safe operating limits (Zhu et al., 2025), providing a new lens for assessing sustainability in arid environments. This draws the attention of policy makers to keep pace with the declining resilience in forest ecosystems, and develop strategies to enhance species richness. In many areas, wherever increased aridity is an issue, integrated strategies of land use plan need to be developed for sustaining ecosystem as a whole.

*Urban environments and environmental justice*: One study from Fujian Province, China, illustrates how urban form and socio‐economic characteristics shape inequality in air pollution exposure (Ling et al., 2025). The population density is associated with worse air quality, and motor vehicle traffic is the most significant source of pollution, with the highest pollution levels found in the most densely populated areas of the city (Wrightson et al., 2025). Alongside evidence from China linking particulate matter pollution to tuberculosis risk, these studies highlight strcutural and socio‐economic drivers of unequal environmental exposures (Ma et al., 2026). By linking urban planning, economic activity, and social segregation with environmental outcomes, the research underscores the importance of integrating environmental justice into urban governance. Studies that integrate environmental, social, and economic perspectives have found that user‐generated content can serve as a supplementary tool for analyzing the dynamics of urban outdoor spaces. This helps facilitate effective urban planning, and thereby promotes the sustainable development of urban societies (Martí et al., 2020).

In summary, the eight contributions reveal several unifying insights into the dynamics of human‐environment interactions. First, land‐use transitions—whether through urban expansion, agricultural intensification, or ecosystem restoration—emerge as central drivers of ecosystem health and carbon dynamics. Second, ecosystem responses are strongly context‐dependent: degraded grasslands, restored forests, arid drylands, and urban environments each exhibit distinct vulnerabilities and management needs, underscoring the importance of site‐specific and scale‐appropriate approaches. Third, the studies collectively advance methodological frontiers, from integrating crop rotation histories and human activity indices to applying planetary boundary frameworks and equity‐based urban assessments, thereby bridging scientific innovation with policy relevance. Most importantly, they demonstrate that safeguarding terrestrial ecosystem health and enriching soil carbon stocks requires not only technical solutions, but also governance systems that integrate ecological, socio‐economic and institutional dimensions (Spotorno et al., 2025).

## 4 Lessons for Science, Practice and Policy

**Figure 1:**
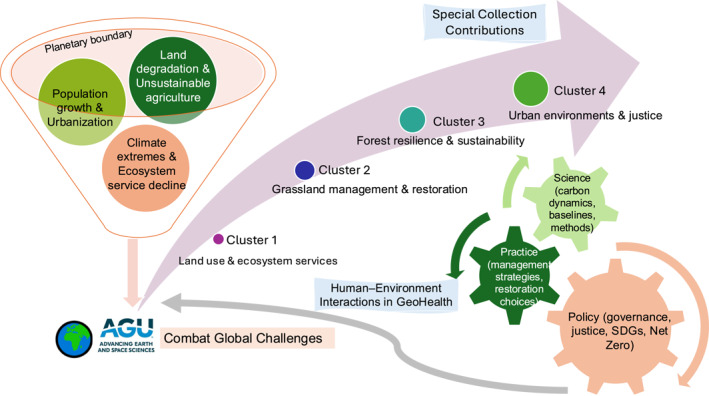
Human–environment interactions in GeoHealth: Pathways from global challenges to science–policy.

Based on the findings from previous studies, this section synthesizes the following key insights. From a scientific perspective, the Special Issue demonstrates the value of integrating biophysical processes with socio‐economic drivers to capture the full complexity of human‐environment interactions. Ecosystem resilience and soil carbon dynamics cannot be understood in isolation, but are mediated by land‐use transitions, restoration practices and climate variability (Doetterl et al., 2025). From a policy standpoint, the contributions highlight the necessity of evidence‐based, context‐specific interventions: quantifying the amount of water resources under the future climate change impact, and subsequently constraining the urban land area based on the water resource carrying capacity while adjusting the configuration of urban landscape—this approach helps to achieve the sustainable development of cities (Liu et al., 2020). These insights provide a roadmap for strengthening human‐environment interactions within the broader GeoHealth and sustainable development agenda (Figure 1).

Beyond their individual contributions, the eight articles collectively deliver special lessons for advancing sustainable management of terrestrial ecosystems. Scientifically, they demonstrate that carbon dynamics are contingent on ecological baselines—whether soils are nutrient‐poor or carbon‐rich, whether forests are species‐poor or diverse—and that restoration and management strategies must be designed with these starting conditions in mind. Practically, these findings show that successful interventions require adaptive, ecosystem‐specific approaches—from improving crop rotation stability to choosing between active and passive restoration, or aligning forest recovery with biodiversity enrichment. At the society level, the local communities need to be integrated with their knowledge and practices to co‐design adaptive strategies and pathways. This may impact the ultimate outcomes at the regional scale. At the policy level, the studies call for governance frameworks that integrate climate adaptation, equity and ecosystem service trade‐offs into decision‐making. While pursuing this, planning should consider the indicators and approaches required in enhancing Geo‐Health sustainability from micro to regional and global level.

## 5 Future Prospects

The GeoHealth framework offers a forward‐thinking platform for advancing research and practice at the interface of ecosystems, climate and human well‐being. Future studies should aim to integrate multi‐scale data‐from remote sensing and field observations to socio‐economic indicators‐into predictive models that can better capture the complexity of human‐environment interactions (Wu et al., 2026). Strengthening cross‐regional and cross‐disciplinary, multi‐stakeholder collaboration is essential, not only for comparing management strategies across diverse ecological and socio‐political contexts, but also for co‐developing adaptive solutions with stakeholders and policymakers. By combining methodological innovation with inclusive governance approaches, the GeoHealth community can effectively contribute to safeguarding ecosystem health, enhancing soil carbon sequestration and supporting the advancement of global climate and sustainability goals.

## Declaration of Generative AI in Scientific Writing

During the preparation of this work, generative AI tools (ChatGPT 5, developed by OpenAI) were used only to improve the clarity of language and polish the writing. The authors reviewed, edited, and approved all content, and take full responsibility for the scientific integrity and originality of the paper.

## Conflict of Interest

The authors declare no conflicts of interest relevant to this study.

## Data Availability

No new data sets or code were generated in this study. The only new research object created is the conceptual diagram presented in the manuscript. This figure was developed by the authors for illustrative purposes and does not rely on underlying data.
